# Albumin and neutrophil combined prognostic grade for predicting overall survival in colorectal cancer: a retrospective cohort study

**DOI:** 10.3389/fonc.2026.1785110

**Published:** 2026-03-12

**Authors:** Xuelian Shi, Guo Tian, Chaoxi Zhou, Jiena Zhou, Haiyan Fu, Chunfu Wan, Xiaoli Xu

**Affiliations:** 1 Department of Painology, The Fourth Hospital of Hebei Medical University, Shijiazhuang, Hebei, China; 2 Department of Medical Record, The Fourth Hospital of Hebei Medical University, Shijiazhuang, Hebei, China; 3 Department of General Surgery, The Fourth Hospital of Hebei Medical University, Shijiazhuang, Hebei, China

**Keywords:** albumin and neutrophil combined prognostic grade, colorectal cancer, inflammatory-nutritional biomarker, overall survival, prognostic nomogram

## Abstract

**Background:**

Despite significant advances in the management of colorectal cancer (CRC), accurate prognostic stratification remains a clinical challenge. Although several inflammation- and nutrition-based prognostic scores have been evaluated in CRC, their clinical utility is often limited by inconsistent cutoff values and overlapping predictive information. While the Glasgow Prognostic Score (GPS) has demonstrated robust prognostic performance in CRC patients, its dependence on C-reactive protein (CRP) measurement may limit its practicality in clinical settings. To address this limitation, this study aimed to investigate the prognostic value of a novel albumin and neutrophil combined prognostic grade (ANPG) system and to develop a clinically applicable nomogram for predicting overall survival (OS) in CRC patients following curative resection.

**Methods:**

A retrospective analysis was conducted on 660 consecutive patients with primary CRC who underwent R0 resection between December 2017 and December 2018. The ANPG was constructed based on preoperative serum albumin levels and neutrophil counts, with optimal cutoff values determined by receiver operating characteristic (ROC) curve analysis. Prognostic factors were identified using univariate and multivariate Cox proportional hazards regression models. A predictive nomogram was developed and internally validated via bootstrap resampling (800 iterations) and time-dependent ROC analysis. Decision curve analysis (DCA) was performed to assess clinical utility.

**Results:**

The median follow-up duration was 2442 days with 108 cancer-specific deaths recorded. The ANPG demonstrated superior discriminative ability (AUC = 0.637, 95% CI: 0.588–0.687, P < 0.001) compared to established inflammatory and nutritional markers, including the neutrophil-to-lymphocyte ratio (NLR), platelet-to-lymphocyte ratio (PLR), systemic immune-inflammation index (SII), fibrinogen-to-albumin ratio (FAR), and fibrinogen-neutrophil-lymphocyte ratio (F-NLR). Kaplan-Meier survival analysis revealed significant OS differences across ANPG grades (Log-rank χ² = 24.423, P < 0.001), with 5-year OS rates of 93.7%, 83.2%, and 74.4% for grades 0, 1, and 2, respectively. Multivariate Cox regression analysis identified ANPG (grade 1 vs. 0: hazard ratio [HR] = 2.190, P = 0.020; grade 2 vs. 0: HR = 3.256, P = 0.001), age (HR = 1.032, P = 0.001), carbohydrate antigen 19-9 (CA19-9) (HR = 1.002, P = 0.003), histological type (HR = 1.954, P = 0.005), and TNM stage as independent prognostic factors. The nomogram incorporating these variables(retaining carcinoembryonic antigen for clinical relevance and model performance) achieved a concordance index of 0.806 (95% CI: 0.788–0.824) with excellent calibration, significantly outperforming TNM staging alone in predictive accuracy. DCA showed greater net benefit than TNM for 5-year OS.

**Conclusions:**

The ANPG score, derived from routinely available laboratory parameters, provides a practical and accessible tool for prognostic stratification in CRC patients. Additionally, the developed nomogram provides a clinically valuable tool for individualized survival prediction and risk stratification in this patient population.

## 1 Introduction

Colorectal cancer (CRC) represents a major global health burden, ranking as the third most frequently diagnosed malignancy and the second leading cause of cancer-related mortality worldwide (1). Despite advances in surgical techniques and adjuvant therapies, disease recurrence or metastasis develops in 20–30% of patients after curative-intent resection, highlighting the necessity for accurate prognostic assessment to guide treatment decisions and postoperative surveillance (2, 3).

The tumor-node-metastasis (TNM) classification system remains the cornerstone of colorectal cancer (CRC) prognostic stratification. However, this anatomical staging approach has inherent limitations in predicting individual patient outcomes, as demonstrated by considerable heterogeneity in clinical progression among patients with identical TNM stages (4–6). This highlights the need to incorporate additional prognostic markers to improve risk assessment accuracy.

Accumulating evidence suggests that cancer-related systemic inflammation and nutritional status play pivotal roles in disease progression and clinical outcomes (7–9). The interplay between chronic inflammation and malnutrition contributes to a tumor-permissive microenvironment, promoting cellular proliferation, local invasion, and distant metastasis (10, 11). Although established inflammatory and nutritional markers—such as the neutrophil-to-lymphocyte ratio (NLR), platelet-to-lymphocyte ratio (PLR), systemic immune-inflammation index (SII), fibrinogen-to-albumin ratio (FAR), and the F-NLR score—have demonstrated prognostic value (12–18), their clinical utility is often limited by inconsistent cutoff values and overlapping predictive information. While the Glasgow Prognostic Score (GPS), which integrates C-reactive protein (CRP) and serum albumin as core indicators, has shown robust prognostic performance in CRC patients with normal preoperative carcinoembryonic antigen (CEA) levels (19). However, its dependence on CRP measurement may limit its practicality in clinical settings where CRP is not routinely assessed.

The ANPG system represents a novel prognostic model that simultaneously assesses nutritional (albumin) and inflammatory (neutrophil) parameters. Serum albumin is a well-established marker of both nutritional status and systemic inflammation, with hypoalbuminemia consistently linked to poor outcomes across various malignancies (20, 21). Neutrophils contribute to tumor-associated inflammation by releasing pro-tumorigenic mediators such as cytokines, chemokines, and reactive oxygen species, which promote tumor progression and immune evasion (22, 23). By integrating these two clinically relevant parameters, the ANPG system provides a more comprehensive assessment of host inflammatory-nutritional status than single biomarkers or existing composite scores. A key advantage of the ANPG system is its reliance on routine preoperative laboratory measurements—serum albumin and neutrophil count—which are widely available, cost-effective, and easy to implement in clinical practice. This simplicity enhances its applicability compared to multi-parameter models (e.g., SII, F-NLR) that require complex calculations or multiple hematological variables, thus supporting broader clinical adoption.

Although previous studies have investigated the prognostic value of the ANPG in non-small cell lung cancer (24), its role in colorectal cancer remains insufficiently characterized. This study aims to: (1) systematically assess the prognostic utility of ANPG in CRC patients following radical resection; (2) quantitatively compare its predictive performance with conventional inflammatory and nutritional biomarkers; and (3) develop and validate a prognostic nomogram incorporating ANPG for individualized survival prediction.

## 2 Materials and methods

### 2.1 Study design and patient selection

This retrospective cohort study was conducted at the Fourth Hospital of Hebei Medical University, including consecutive patients who underwent curative-intent surgery for primary CRC between December 2017 and December 2018. This study was approved by the Ethics Committee of the Fourth Hospital of Hebei Medical University (approval No. 2025KS056) and conducted in accordance with the Declaration of Helsinki. Owing to its retrospective design, the requirement for informed consent was waived.

Eligibility criteria included: (1) histologically confirmed primary colorectal cancer (CRC), including adenocarcinoma or mucinous adenocarcinoma; (2) complete R0 resection with microscopically negative margins; (3) documented preoperative serum albumin and neutrophil counts measured within 7 days prior to surgery; (4) availability of comprehensive follow-up data; and (5) for stage IVA (M1a) patients, synchronous single-organ metastasis (hepatic or pulmonary) with R0 resection of both primary and metastatic lesions, absence of peritoneal or multi-organ dissemination, and completion of 6 months of standard adjuvant chemotherapy.

Exclusion criteria were: (1) stage IVB/IVC disease or non-R0 resection; (2) concomitant malignancies, significant cardiovascular comorbidities, or end-stage organ dysfunction; (3) major postoperative complications (e.g., anastomotic leak complicated by septic shock); (4) prior neoadjuvant therapy or use of medications known to affect albumin levels or hematological parameters (e.g., corticosteroids, nonsteroidal anti-inflammatory drugs); (5) death from non-oncologic causes or loss to follow-up.

### 2.2 Data-access and privacy statement

To further ensure patient privacy, we clarify that all records were fully de-identified before analysis; the authors had no access to any information that could identify individual participants during or after data collection. The clinical dataset was extracted from the electronic medical record system on 15 January 2024 for research purposes. The requirement for informed consent was waived by the Ethics Committee of the Fourth Hospital of Hebei Medical University (approval No. 2025KS056).

### 2.3 Data collection and variable definition

Clinical and pathological variables were systematically extracted from electronic medical records, including demographic characteristics (age, sex), tumor-related features (TNM stage according to the 8th edition of the American Joint Committee on Cancer [AJCC] staging system, histological subtype, perineural invasion [PNI], lymphovascular invasion [LVI]), therapeutic interventions (postoperative adjuvant chemotherapy), and preoperative laboratory markers (serum albumin, neutrophil count, lymphocyte count, platelet count, fibrinogen, carcinoembryonic antigen [CEA], and carbohydrate antigen 19–9 [CA19-9]).

### 2.4 The following inflammatory and nutritional indices were calculated

Neutrophil-to-lymphocyte ratio (NLR): defined as neutrophil count divided by lymphocyte count. Platelet-to-lymphocyte ratio (PLR): calculated as platelet count divided by lymphocyte count. Systemic immune-inflammation index (SII): computed as (platelet count × neutrophil count)/lymphocyte count. Fibrinogen-to-albumin ratio (FAR): determined as fibrinogen concentration divided by albumin concentration, and patients were classified into two groups based on predefined cutoff: FAR_cat = 0 (FAR ≤ 0.091734) and FAR_cat = 1 (FAR > 0.091734). Fibrinogen-neutrophil-lymphocyte ratio (F-NLR) score: patients were classified into three groups based on predefined cutoffs (fibrinogen ≥3.595 g/L and NLR ≥3.0157): score 2 (both elevated), score 1 (one elevated), or score 0 (neither elevated).

### 2.5 Albumin and neutrophil combined prognostic grade system

The ANPG is a categorical grading system, not a continuous score or mathematical ratio. It classifies patients into three grades based on the combined status of two binary variables-–-preoperative serum albumin and neutrophil count values. Optimal cutoffs were identified through receiver operating characteristic (ROC) curve analysis, with the maximum Youden index (sensitivity + specificity − 1) used to determine thresholds for predicting overall survival. The optimal cutoffs were 42.35 g/L for albumin and 3.755×10^9^/L for neutrophils.

### 2.6 Patients were categorized into three ANPG grades

Grade 0 (well-nourished with mild inflammation): albumin ≥42.35 g/L and neutrophil count <3.755×10^9^/L. Grade 1 (single nutritional or inflammatory abnormality): either albumin ≥42.35 g/L and neutrophil count ≥3.755×10^9^/L (well-nourished with systemic inflammation), or albumin <42.35 g/L and neutrophil count <3.755×10^9^/L (malnourished without significant inflammation). Grade 2 (malnourished with severe inflammation): albumin <42.35 g/L and neutrophil count ≥3.755×10^9^/L.

### 2.7 Follow-up and study endpoint

Postoperative follow-up was performed at 3–6-month intervals during the first 2 years and annually thereafter. Follow-up assessments included outpatient clinic visits, telephone interviews, and review of electronic medical records. The primary endpoint was overall survival (OS), defined as the time from date of surgery to date of death from any cause or last known follow-up. The study follow-up period ended on December 16, 2024.

### 2.8 Statistical analysis

Statistical analyses were performed using SPSS version 25.0 (IBM Corp., Armonk, NY, USA) and R version 4.2.1 (R Foundation for Statistical Computing, Vienna, Austria), with the following packages: survival (v3.3.1), rms (v6.3-0), pROC, and ggplot2. Continuous variables were summarized as median (interquartile range [IQR]) or mean ± standard deviation (SD), while categorical variables were presented as frequency (percentage). Receiver operating characteristic (ROC) curve analysis was used to assess the discriminative performance of biomarkers, with area under the curve (AUC) values and corresponding 95% confidence intervals (CIs) calculated. Comparison of AUCs between different markers was conducted using DeLong’s test.

Survival analysis was carried out using the Kaplan-Meier method, and group differences were evaluated by the log-rank test. Univariate and multivariate Cox proportional hazards regression models were applied to identify independent prognostic factors. Variables with a univariate P value < 0.10 were included in the multivariate model. Multicollinearity was assessed using the variance inflation factor (VIF), and a VIF < 4 was considered acceptable.

A nomogram was constructed based on the results of multivariate Cox regression using the rms package. Model discrimination was evaluated by the concordance index (C-index) and time-dependent ROC (tdROC) analysis. Calibration curves were generated to assess the agreement between predicted and observed survival probabilities, and internal validation was performed using bootstrap resampling (800 iterations, 100 samples per iteration). Clinical utility was further evaluated through decision curve analysis (DCA).

All statistical tests were two-sided, and a P value < 0.05 was considered statistically significant.

## 3 Results

### 3.1 Patient characteristics

From December 2017 to December 2018, a total of 660 consecutive eligible patients were enrolled (**Table 1**). The median age was 61 years (IQR: 53–68), and 353 (53.5%) were male. TNM stage distribution was as follows: stage I (n = 102, 15.5%), stage II (n = 298, 45.2%), stage III (n = 249, 37.7%), and stage IVA (n = 11, 1.7%). The majority of tumors were conventional adenocarcinoma (n = 572, 86.7%), while mucinous adenocarcinoma accounted for 13.3% (n = 88). Postoperative adjuvant chemotherapy was administered to 407 patients (61.7%).

**Table 1:** Baseline clinical characteristics of CRC patients stratified by ANPG grade.

Characteristic	Total cases (n=660)	ANPG grade 0 (n=175)	ANPG grade 1 (n=309)	ANPG grade 2 (n=176)	P-value
Age [years, M (P25, P75)]]	61(53,68)	60 (52,65)	61 (54,68)	64(55.75,70)	0.001
Gender, n (%)					0.349
Male	353(53.5)	86 (13%)	173 (26.2%)	94 (14.2%)	
Female	307(46.5)	89 (13.5%)	136 (20.6%)	82 (12.4%)	
TNM stage, n (%)					0.200
Stage I	102(15.5)	38 (5.8%)	44 (6.7%)	20 (3%)	
Stage II	298(45.2)	76 (11.5%)	140 (21.2%)	82 (12.4%)	
Stage III	249(37.7)	59 (8.9%)	120 (18.2%)	70 (10.6%)	
Stage IVA	11(1.7)	2 (0.3%)	5 (0.8%)	4 (0.6%)	
Histological type, n (%)					0.379
Conventional Adenocarcinoma	572(86.7)	157 (23.8%)	265 (40.2%)	150 (22.7%)	
Mucinous Adenocarcinoma	88(13.3)	18 (2.7%)	44 (6.7%)	26 (3.9%)	
Perineural invasion (PNI), n (%)					0.352
No	499(75.6)	131 (19.8%)	228 (34.5%)	140 (21.2%)	
Yes	161(24.4)	44 (6.7%)	81 (12.3%)	36 (5.5%)	
Lymphovascular invasion (LVI), n (%)					0.736
No	646(97.9)	172 (26.1%)	301 (45.6%)	173 (26.2%)	
Yes	14(2.1)	3 (0.5%)	8 (1.2%)	3 (0.5%)	
Postoperative adjuvant chemotherapy, n (%)					0.682
Yes	407(61.7)	105 (15.9%)	196 (29.7%)	106 (16.1%)	
No	253(38.3)	70 (10.6%)	113 (17.1%)	70 (10.6%)	

As of the final follow-up date (December 16, 2024), the median follow-up duration was 2442 days (IQR: 2117–2537). During this period, 108 tumor-related deaths were recorded, resulting in an overall censoring rate of 83.6%.

### 3.2 ANPG distribution and baseline characteristics

According to the ANPG classification, the cohort included 175 patients (26.5%) classified as grade 0, 309 (46.8%) as grade 1, and 176 (26.7%) as grade 2. Baseline characteristics stratified by ANPG grade are shown in **Table 1**. Patients with higher ANPG grades were significantly older (P = 0.001). No significant differences were observed across the three groups in terms of sex, TNM stage, histological type, perineural invasion, lymphovascular invasion, or receipt of adjuvant chemotherapy (all P > 0.05).

### 3.3 Discriminative performance of ANPG compared to other biomarkers

**Figure 1:**
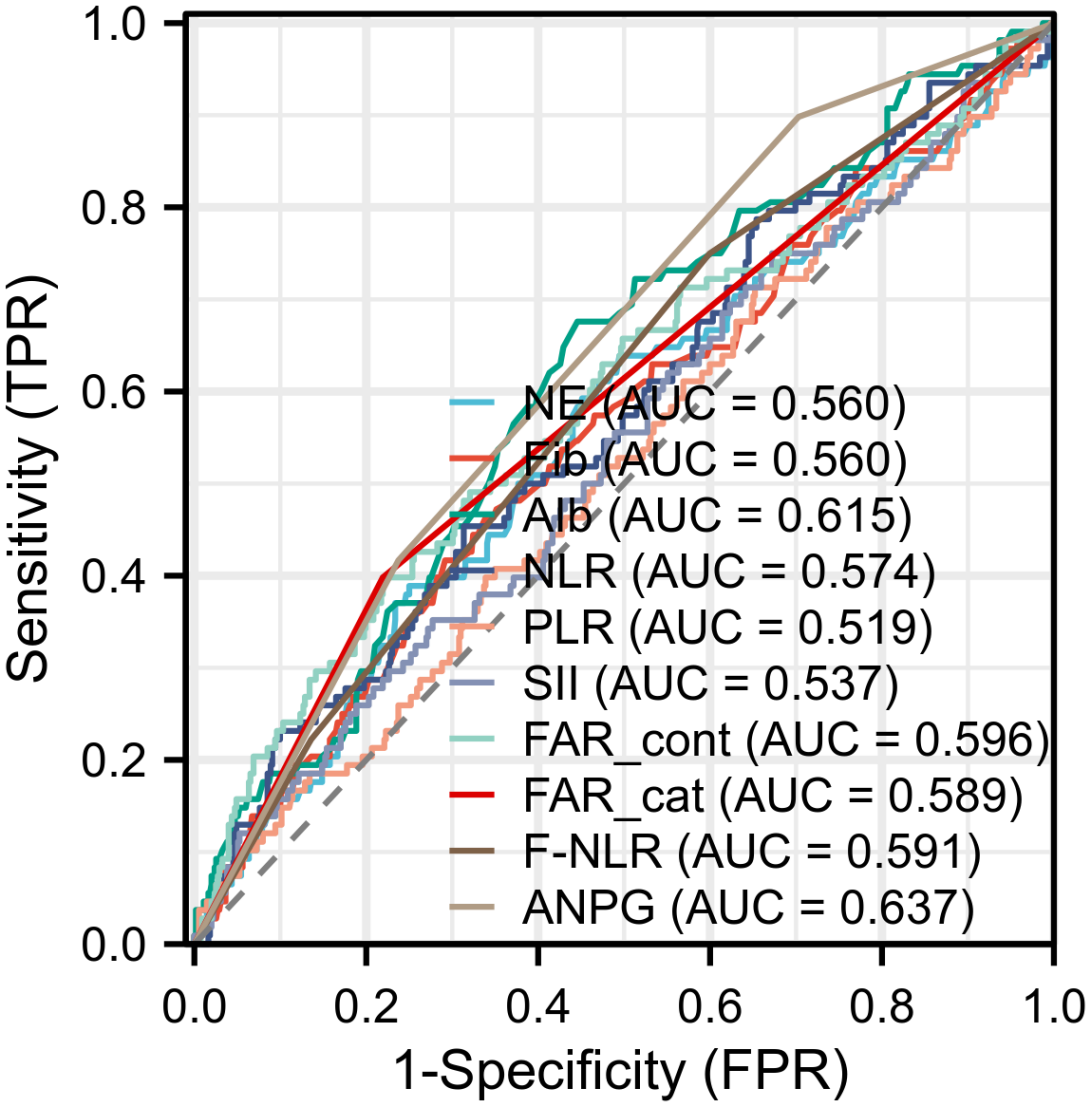
Receiver operating characteristic (ROC) curve analysis demonstrates the comparative prognostic performance of the albumin and neutrophil combined prognostic grade (ANPG) system versus other inflammatory and nutritional biomarkers in predicting overall survival (OS) among colorectal cancer (CRC) patients. The ANPG system exhibited superior discriminative ability, achieving the highest area under the curve (AUC) value of 0.637, which was statistically significantly greater than those of neutrophil-to-lymphocyte ratio (NLR, 0.574), platelet-to-lymphocyte ratio (PLR, 0.519), systemic immune-inflammation index (SII, 0.537), continuous fibrinogen-to-albumin ratio (FAR_cont, 0.596), categorical fibrinogen-to-albumin ratio (FAR_cat, 0.589), and fibrinogen-NLR (F-NLR, 0.591) (DeLong’s test, all P < 0.05).

Receiver operating characteristic (ROC) curve analysis was performed to evaluate the discriminative ability of ANPG and other inflammatory/nutritional markers in predicting overall survival (OS) (**Table 2**; **Figure 1**). Among all assessed biomarkers, ANPG exhibited the highest area under the curve (AUC) value (0.637, 95% CI: 0.588–0.687, P < 0.001). DeLong’s test demonstrated that the AUC of ANPG was significantly greater than those of the neutrophil-to-lymphocyte ratio (NLR) (AUC = 0.574, P = 0.047), platelet-to-lymphocyte ratio (PLR) (AUC = 0.519, P = 0.002), systemic immune-inflammation index (SII) (AUC = 0.537, P = 0.001), and F-NLR score (AUC = 0.591, P = 0.049). For the fibrinogen-to-albumin ratio (FAR), the continuous variable (FAR_cont, log-transformed using the natural logarithm (Ln) to account for non-normal distribution), yielded an AUC of 0.596, whereas the categorical variable (FAR_cat) resulted in an AUC of 0.589 (95% CI: 0.540–0.639, P = 0.003). Additionally, ANPG showed superior predictive performance compared to albumin (AUC = 0.615) and neutrophil count (AUC = 0.560) as individual parameters.

**Table 2:** Results of ROC curve analysis for prognostic indicators in CRC patients.

Indicator	Confidence interval (CI)	Sensitivity (%)	Specificity (%)	Youden index	Cut-off value	P-value
NE	0.498 – 0.622	0.639	0.505	0.144	3.755	0.049
Fib	0.499–0.621	0.417	0.708	0.125	3.595	0.049
AIb	0.558 – 0.673	0.676	0.554	0.230	42.35	<0.001
NLR	0.514 – 0.634	0.454	0.687	0.140	3.016	0.015
PLR	0.458 – 0.580	0.713	0.348	0.061	145.430	0.524
SII	0.476 – 0.598	0.750	0.328	0.078	492.020	0.220
FAR	0.534–0.659	0.398	0.781	0.179	0.092	0.002
FAR_cont	0.534–0.659	0.398	0.781	0.179	-2.389	0.002
FAR_cat	0.540–0.639	0.398	0.781	0.179	0.500	0.003
F-NLR	0.538 – 0.644	0.750	0.400	0.150	0.500	0.003
ANPG	0.588 – 0.687	0.898	0.297	0.195	0.500	<0.001

NE, Neutrophils; AIb, Albumin; NLR, Neutrophil-to-Lymphocyte Ratio; PLR, Platelet-to-Lymphocyte Ratio; SII, Systemic Immune-Inflammation Index; FAR, Fibrinogen-to-albumin ratio (FAR); FAR_cont, Fibrinogen-to-albumin ratio (FAR) Continuous variable after natural logarithm (Ln) transformation; FAR_cat, Fibrinogen-to-albumin ratio (FAR) Categorical; F-NLR, Fibrinogen-Neutrophil-Lymphocyte Ratio; ANPG, Albumin-Neutrophil Combined Prognostic Grade.

The original FAR threshold of 0.091734 (rounded to 0.092 in the table to maintain three significant figures) corresponds to a FAR_cont value of −2.389, as these values represent equivalent thresholds [ln(0.091734) ≈ −2.389]. The categorical variable FAR_cat (0/1) is based on this cutoff and was used in the final multivariate model.

### 3.4 ANPG and overall survival

**Figure 2:**
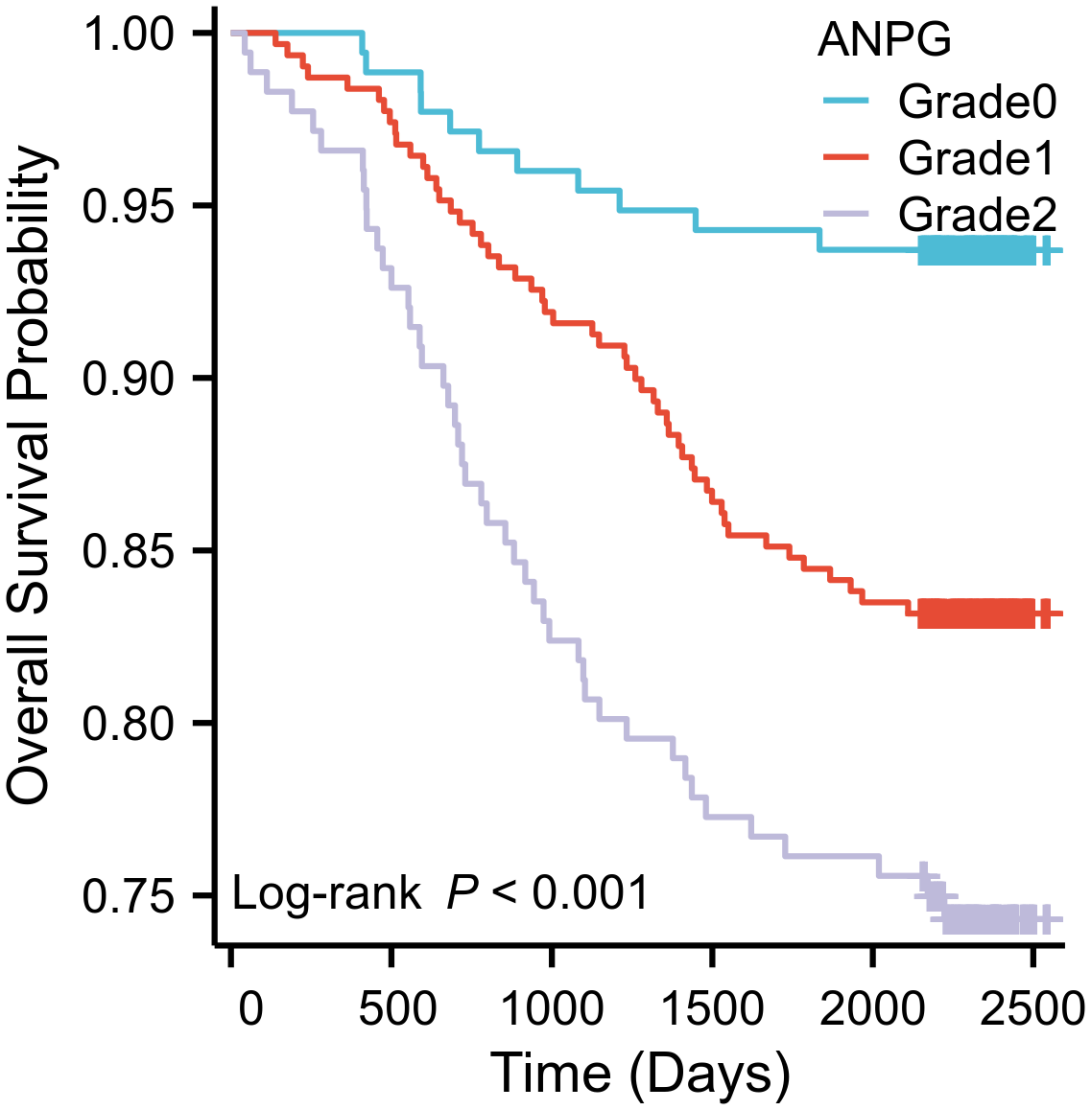
Kaplan-Meier survival analysis stratified by ANPG grade in a cohort of 660 CRC patients revealed significant survival differences. The 5-year OS rates were 93.7% (n=175) for Grade 0, 83.2% (n=309) for Grade 1, and 74.4% (n=176) for Grade 2. A significant inverse association was observed between ANPG grade and OS (log-rank χ²=24.423, P<0.001), with all pairwise comparisons demonstrating statistical significance (P<0.05).

Kaplan-Meier survival analysis revealed significant differences in overall survival (OS) across the three ANPG grade groups (log-rank χ² = 24.423, P < 0.001) (**Figure 2**). Mean OS durations were 2442.743 ± 31.217 days for grade 0, 2296.343 ± 33.854 days for grade 1, and 2116.641 ± 59.011 days for grade 2. The corresponding 5-year OS rates were 93.7%, 83.2%, and 74.4%, respectively. Pairwise comparisons showed statistically significant differences between all ANPG grade groups (all P < 0.05).

### 3.5 Univariate and multivariate cox regression analysis

Univariate Cox regression analysis identified several factors significantly associated with overall survival (OS) (**Table 3**), including age, carcinoembryonic antigen (CEA), carbohydrate antigen 19-9 (CA19-9), systemic immune-inflammation index (SII), fibrinogen-to-neutrophil-lymphocyte ratio (F-NLR), albumin and neutrophil combined prognostic grade (ANPG), TNM stage, histological type, and postoperative adjuvant chemotherapy (all P < 0.10).

**Table 3:** Univariate and Multivariate Cox regression analysis of OS in CRC patients.

Variables	Univariate analysis		Multivariate analysis	
	HR(95% CI)	P-value	HR(95% CI)	P-value
Age (per 1-year increase)	1.033 (1.014 - 1.053)	< 0.001	1.032 (1.013~1.052)	0.001
Gender(Male vs Female)	0.891 (0.611 - 1.299)	0.548		
TNM Stage		<0.001		
Stage II vs Stage I	2.705 (0.812 - 9.008)	0.105	1.837 (0.522 - 6.466)	0.343
Stage III vs Stage I	12.185 (3.842 - 38.644)	< 0.001	8.655(2.563 - 29.222)	< 0.001
Stage IVA vs Stage I	31.848(8.224-123.324)	< 0.001	22.896(5.344- 98.102)	< 0.001
Histological Type (Mucinous adenocarcinoma vs Conventional adenocarcinoma)	2.170 (1.387 - 3.394)	< 0.001	1.954 (1.222 - 3.124)	0.005
Perineural Invasion(Yes vs No)	1.405 (0.933 - 2.116)	0.103		
Lymphovascular Invasion (Yes vs No)	1.728 (0.636 - 4.690)	0.283		
Postoperative Adjuvant Chemotherapy (Yes vs No)	1.843 (1.198 - 2.834)	0.005	1.120 (0.696 - 1.803)	0.641
CEA(per 1 ng/mL increase)	1.009 (1.006 - 1.012)	< 0.001	1.004 (1.000 - 1.008)	0.075
CA19-9(per 1 U/mL increase)	1.003 (1.002 - 1.004)	< 0.001	1.002 (1.001-1.004)	0.003
CA724	1.004 (0.998 - 1.011)	0.172		
Neutrophil-to-Lymphocyte Ratio (NLR)	1.055(0.989 - 1.126)	0.105		
Platelet-to-Lymphocyte Ratio (PLR)	1.001(0.999 - 1.003)	0.244		
Systemic Immune-Inflammation Index (SII)	1.000 (1.000 - 1.000)	0.064	1.000 (0.999 - 1.000)	0.131
Fibrinogen-to-albumin ratio (FAR)_cont	3.564 (1.696 - 7.489)	< 0.001		
Fibrinogen-to-albumin ratio (FAR)_cat	2.201(1.497 - 3.235)	< 0.001	1.164 (0.721 - 1.879)	0.534
Fibrinogen-to-Neutrophil Lymphocyte Ratio (F-NLR)(1 vs 0)	1.758(1.112 - 2.779)	0.016	1.266 (0.771 - 2.080)	0.352
Fibrinogen-to-Neutrophil Lymphocyte Ratio (F-NLR)(2 vs 0)	2.441(1.408 - 4.230)	0.001	1.513 (0.714 - 3.208)	0.280
ANPG(1 vs 0)	2.797(1.460 - 5.361)	0.002	2.190 (1.131 - 4.240)	0.020
ANPG(2 vs 0)	4.593(2.376 - 8.881)	< 0.001	3.256 (1.576 - 6.727)	0.001

Of the 253 patients who did not undergo chemotherapy, 102 (40.3%) were classified as stage I, for whom adjuvant therapy was not indicated according to NCCN guidelines. The initial univariate analysis revealed a significant association between adjuvant chemotherapy and elevated mortality risk (HR = 1.843, 95% CI: 1.198–2.834, P = 0.005). However, this association was no longer significant after multivariate adjustment (HR = 1.120, 95% CI: 0.696–1.803, P = 0.641), indicating confounding by indication. Specifically, patients with advanced-stage disease (stage III) were more likely to receive chemotherapy (48.6% in the chemotherapy group vs. 20.2% in the non-chemotherapy group) and inherently exhibited worse prognoses. Further details are provided in the Results and Discussion sections.

Notably, both the continuous (FAR_cont; HR = 3.564, 95% CI: 1.696–7.489, P < 0.001) and categorical (FAR_cat; HR = 2.201, 95% CI: 1.497–3.235, P < 0.001) forms of the fibrinogen-to-albumin ratio (FAR) were significantly associated with OS in univariate analysis. To minimize multicollinearity due to inclusion of both FAR variants and to enhance clinical applicability, only the categorical FAR variable (FAR_cat) was included in the multivariate Cox proportional hazards regression model. However, after adjustment for ANPG, TNM stage, age, CA19-9, CEA, and histological type, FAR_cat no longer retained statistical significance (P = 0.534). This suggests that when ANPG—a more robust composite inflammatory-nutritional marker—was included in the model, FAR did not independently predict OS in this patient cohort.

Multivariate Cox proportional hazards regression analysis (**Table 3**) identified several independent prognostic factors (all variance inflation factors <4, indicating acceptable multicollinearity): ANPG (grade 1 vs. 0: HR = 2.190, 95% CI: 1.131–4.240, P = 0.020; grade 2 vs. 0: HR = 3.256, 95% CI: 1.576–6.727, P = 0.001), age (per 1-year increase: HR = 1.032, 95% CI: 1.013–1.052, P = 0.001), CA19-9 (per 1 U/mL increase: HR = 1.002, 95% CI: 1.001–1.004, P = 0.003), histological subtype (mucinous adenocarcinoma vs. conventional adenocarcinoma: HR = 1.954, 95% CI: 1.222–3.124, P = 0.005), and TNM stage (stage III vs. I: HR = 8.655, 95% CI: 2.563–29.222, P < 0.001; stage IVA vs. I: HR = 22.896, 95% CI: 5.344–98.102, P < 0.001) were identified as independent prognostic factors. Although CEA did not achieve statistical significance in the final multivariate model (P = 0.075), it was retained due to its established clinical relevance and borderline significant association in univariate analysis (HR = 1.009, 95% CI: 1.006–1.012, P < 0.001). CEA was incorporated as a continuous variable in the nomogram, as preliminary analyses failed to identify an optimal cutoff with adequate discriminative performance (AUC = 0.582, P = 0.012). Furthermore, univariate Cox regression analysis demonstrated a significant association between adjuvant chemotherapy and overall survival (HR = 1.843, 95% CI: 1.198–2.834, P = 0.005). However, after adjusting for variables with P<0.10 in univariate analysis by incorporating them into the multivariate Cox model, the significant association between adjuvant chemotherapy and survival outcomes was abolished (HR = 1.120, 95% CI: 0.696–1.803, P = 0.641).

The proportional hazards assumption was satisfied for all covariates in the multivariate model (global test: χ² = 18.941, P = 0.167).

### 3.6 Forest plot analysis

**Figure 3:**
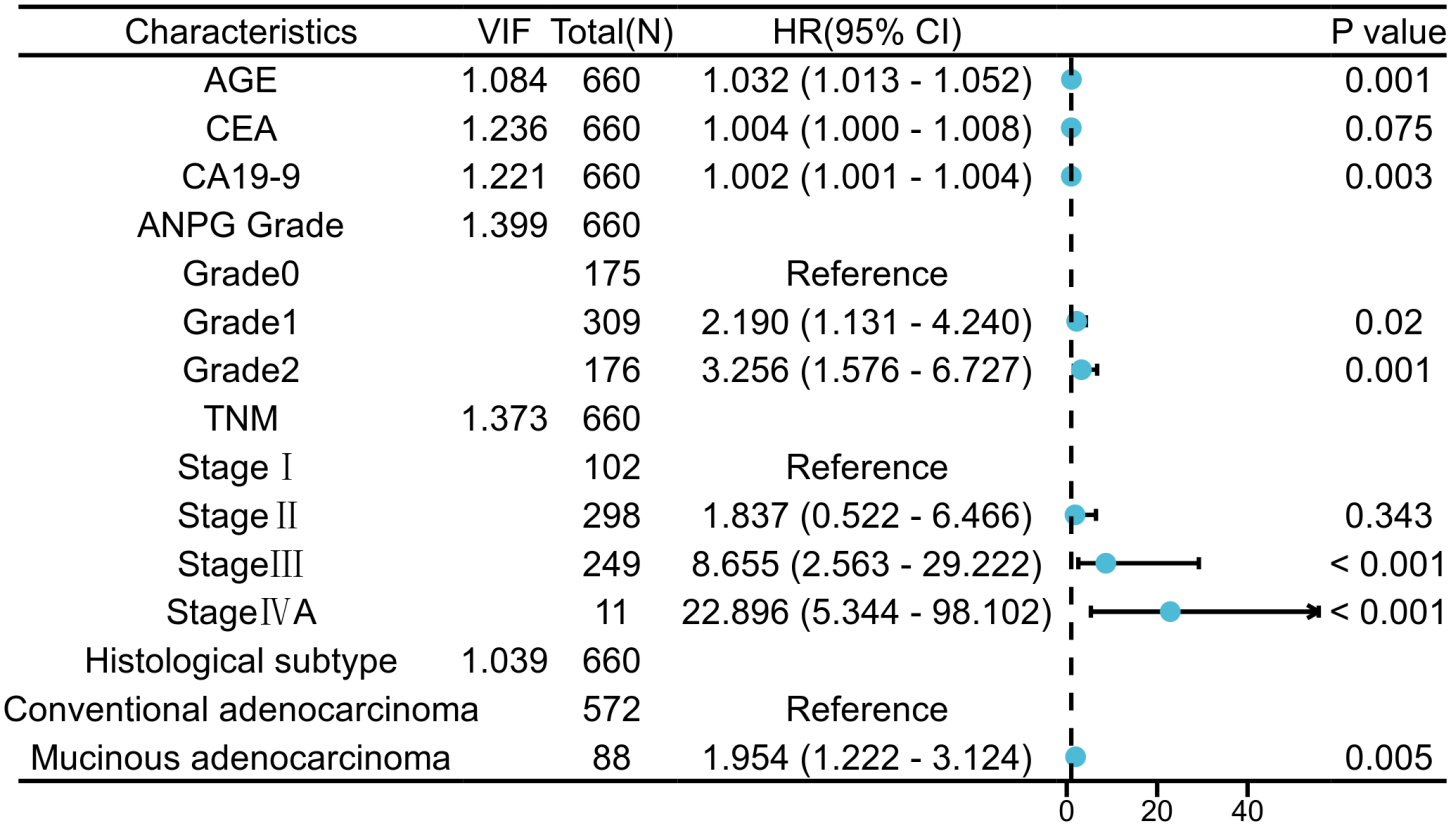
Multivariate Cox proportional hazards regression analysis identified independent prognostic factors for CRC. Notable hazard ratios (95% confidence intervals) included: TNM stage IVA versus I (22.896, 5.344-98.102), ANPG Grade 2 versus Grade 0 (3.256, 1.576-6.727), and age as a continuous variable (1.032 per 1-year increase, 1.013-1.052).

A forest plot was constructed to visualize the effect sizes of the independent prognostic factors (**Figure 3**). The results revealed a hierarchical risk stratification, with TNM stage IVA conferring the highest mortality risk (HR = 22.896), followed by stage III (HR = 8.655). ANPG exhibited a graded increase in risk from grade 0 to grade 2, while age, CA19-9, and histological subtype contributed additional prognostic discrimination.

### 3.7 Nomogram development and validation

#### 3.7.1 Construction of the nomogram model and variable selection

**Figure 4:**
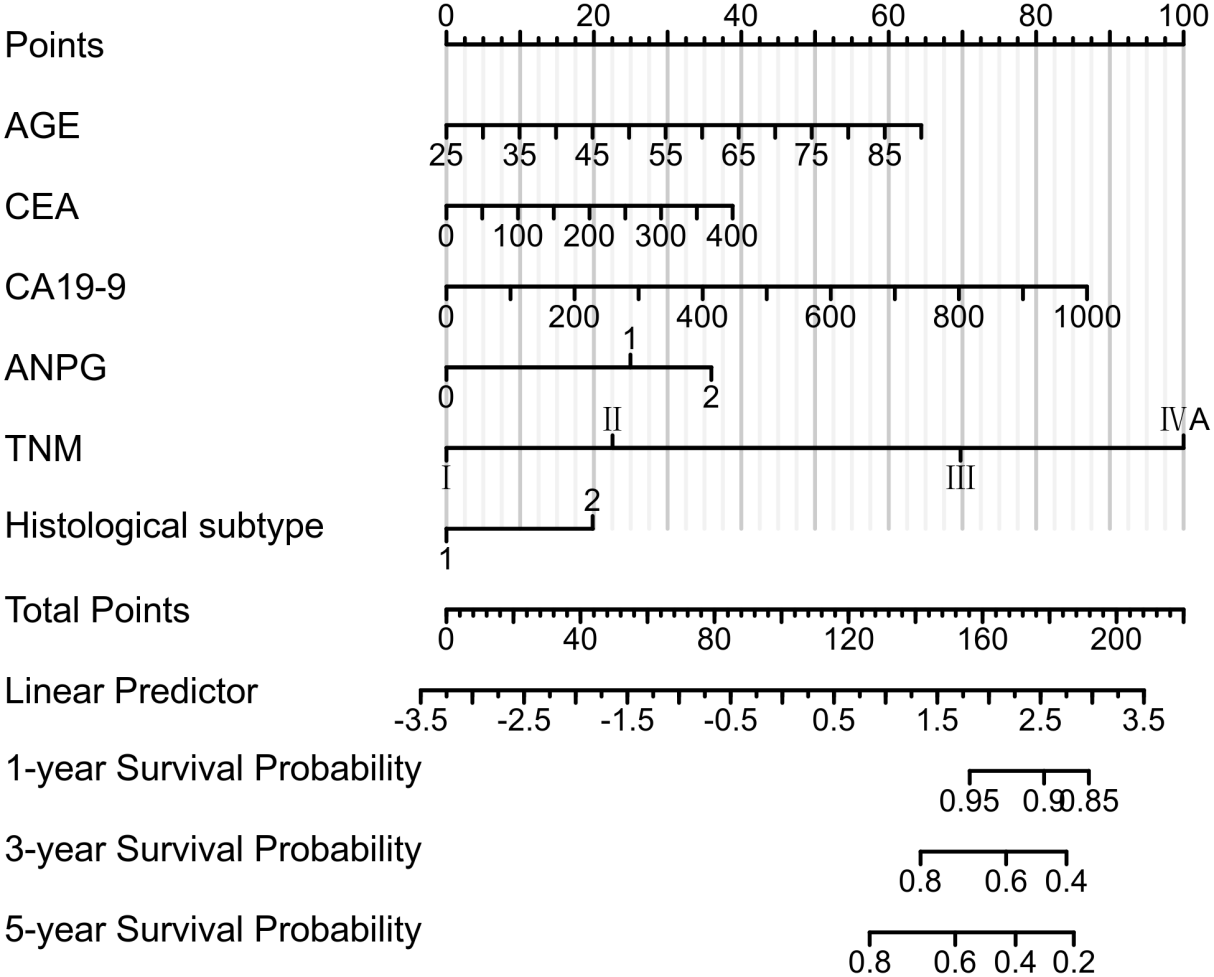
A prognostic nomogram incorporating ANPG, age, carbohydrate antigen 19-9 (CA19-9), carcinoembryonic antigen (CEA), histological type, and TNM stage was developed to predict 1-, 3-, and 5-year OS following radical resection in CRC patients. The model demonstrated excellent discriminative capacity, with a concordance index (C-index) of 0.806 (95% CI 0.788-0.824).

A nomogram was developed to predict 1-year, 3-year, and 5-year overall survival (OS) probabilities in patients with colorectal cancer, incorporating six key variables: ANPG, age, CA19-9, CEA, histological type, and TNM stage (**Figure 4**). Univariate Cox regression analysis demonstrated that CEA was significantly associated with OS (HR = 1.009, 95% CI: 1.006–1.012, P < 0.001). Model comparison showed that inclusion of CEA improved the concordance index (C-index) to 0.806 (95% CI: 0.788–0.824), compared to 0.800 (95% CI: 0.782–0.818) when excluded. Therefore, CEA was retained in the final model.

#### 3.7.2 Validation of model performance

**Figure 5:**
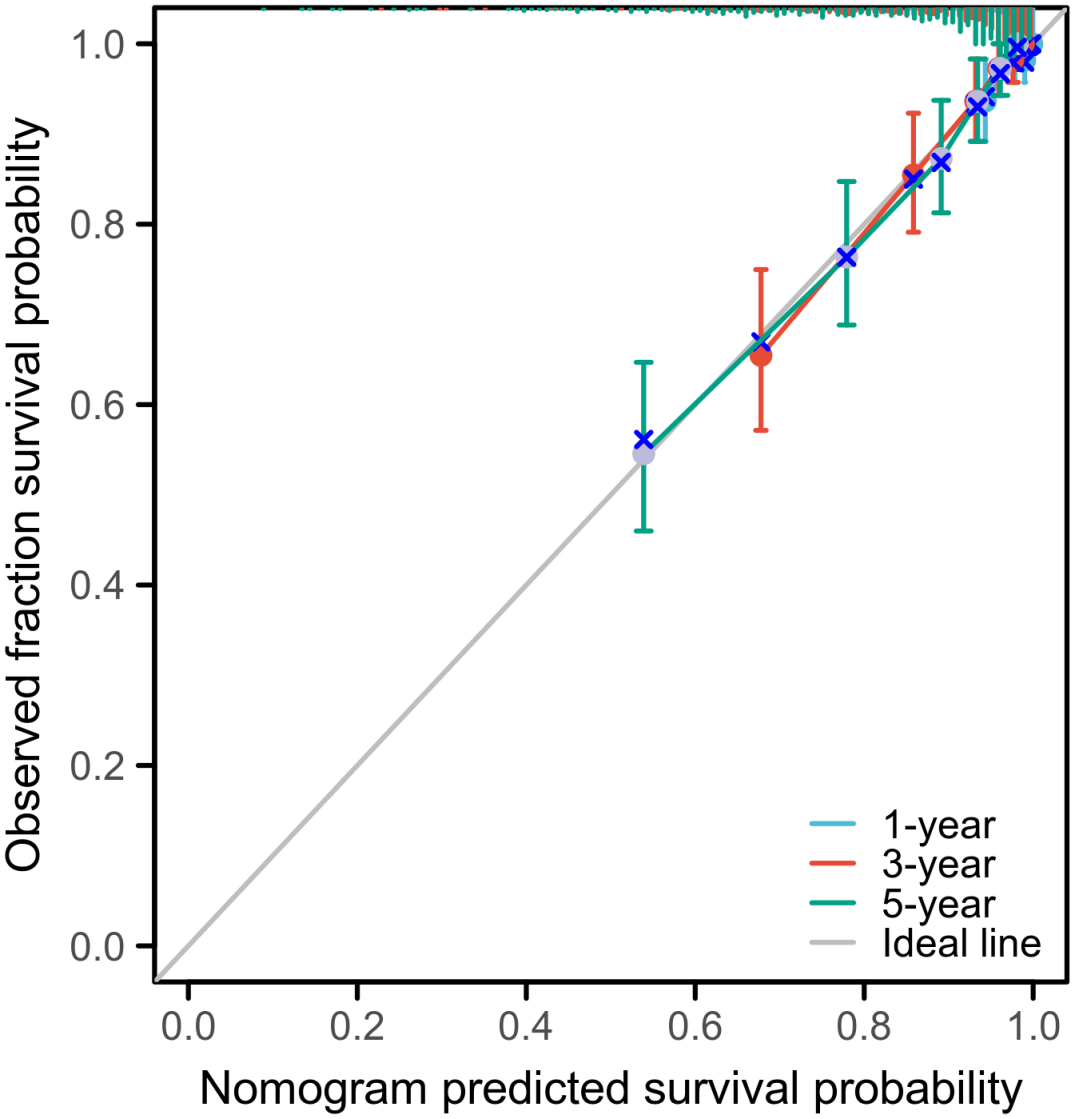
Calibration analysis of the nomogram for 1-, 3-, and 5-year OS prediction showed good agreement between predicted and observed survival probabilities. Internal validation using bootstrap resampling (800 iterations with 100 samples per iteration) confirmed the model’s reliability.

The nomogram demonstrated strong discriminative ability, with a C-index of 0.806 (95% CI: 0.788–0.824). Calibration plots showed excellent agreement between predicted and observed 1-year, 3-year, and 5-year OS probabilities (**Figure 5**). Internal validation using 800 bootstrap resampling iterations (100 samples per iteration) confirmed the robustness and stability of the model.

#### 3.7.3 Time-dependent receiver operating characteristic analysis

**Figure 6:**
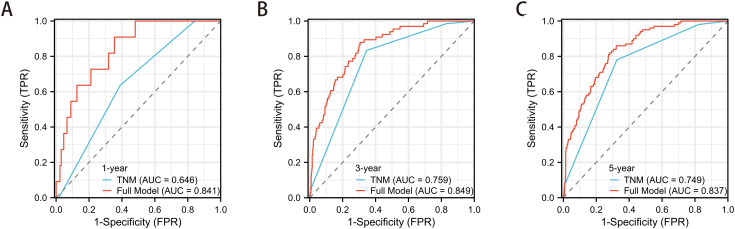
Time-dependent ROC analysis comparing the prognostic performance of the nomogram versus TNM staging system revealed superior discriminative ability of the nomogram for **(A)** 1-year (AUC: 0.841 vs 0.646, P<0.05), **(B)** 3-year (AUC: 0.849 vs 0.759, P<0.05), and **(C)** 5-year (AUC: 0.837 vs 0.749, P<0.05) OS prediction.

Time-dependent ROC analysis was performed to evaluate the dynamic predictive accuracy of the nomogram. The area under the curve (AUC) values for 1-year, 3-year, and 5-year OS predictions were 0.841, 0.849, and 0.837, respectively, all significantly higher than those of the conventional TNM staging system (AUCs: 0.646, 0.759, and 0.749; all P < 0.05) (**Figures 6A-C**).

#### 3.7.4 Decision curve analysis

**Figure 7:**
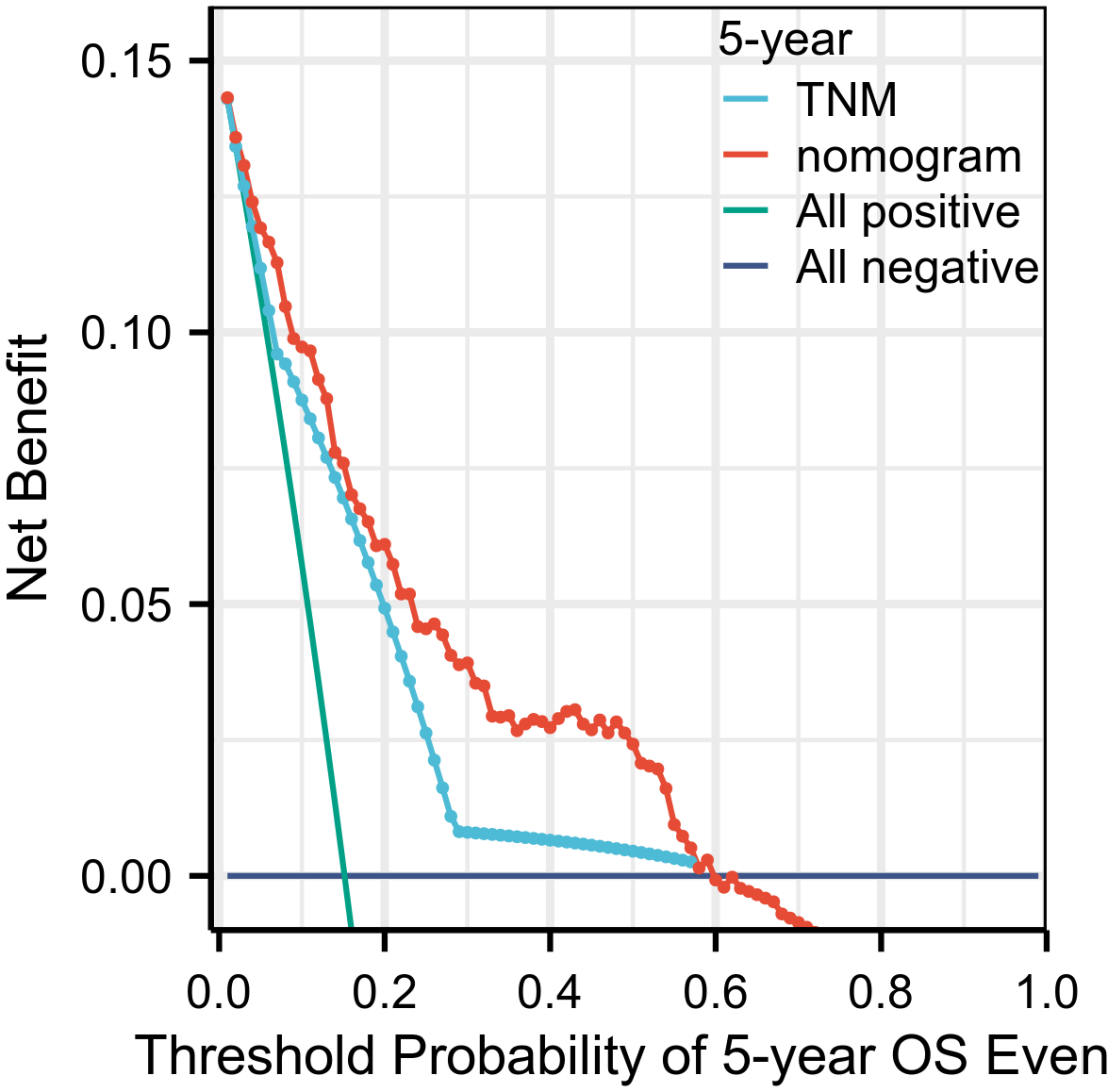
Decision curve analysis demonstrated the clinical utility of the nomogram across a wide range of threshold probabilities, showing consistently higher net benefit compared to both the TNM staging system and default strategies (treat-all or treat-none approaches).

To assess clinical utility, decision curve analysis (DCA) was conducted to quantify the net benefit of the nomogram for predicting 5-year OS across a range of threshold probabilities (0–1.0) (**Figure 7**). Within the clinically meaningful range (0.05–0.70), the nomogram consistently provided greater net benefit than TNM staging alone. Beyond 0.70, the net benefits declined for both strategies, though the nomogram remained superior. These findings support the enhanced clinical applicability of the proposed nomogram over traditional TNM staging.

## 4 Discussion

This study establishes the preoperative albumin and neutrophil combined prognostic grade (ANPG) system as an independent and robust prognostic indicator for patients with colorectal cancer (CRC) undergoing radical resection. Our results demonstrate that ANPG exhibits superior discriminative ability compared to conventional inflammatory and nutritional biomarkers, and its integration into a nomogram significantly enhances individualized survival prediction beyond the traditional TNM staging system.

Our findings are consistent with the previous study and further extend the clinical value of ANPG. Sun et al. (24) first reported the prognostic utility of the ANPG in non-small cell lung cancer, showing improved predictive performance over single parameters(albumin or neutrophil count). Our study extends these observations to colorectal cancer (CRC), establishing ANPG’s consistent prognostic value across different malignant tumors. Wang et al. (25) previously reported that the preoperative Albumin-Neutrophil-to-Lymphocyte Ratio Score (ANS)—incorporating both albumin and neutrophil-to-lymphocyte ratio (NLR)—could predict survival and postoperative complications, postoperative complications, and health-related quality of life in elderly CRC patients. Notably, ANPG differs fundamentally from ANS by utilizing absolute neutrophil count rather than NLR as the inflammatory marker, thereby simplifying computation while preserving prognostic accuracy—an alignment with our objective of enhancing clinical feasibility. In contrast to the Glasgow Prognostic Score (GPS) based on C-reactive protein and albuminas reported in Ishizuka M’s study (19), our model employs neutrophil count as a practical alternative to CRP. By relying only on routine blood tests without additional inflammatory markers, the ANPG shows superior practical utility in primary healthcare settings and is more feasible for widespread implementation in centers with limited laboratory resources where CRP is not routinely measured. The discriminative performance of ANPG (AUC = 0.637) proves comparable to classical prognostic scores, indicating that routine laboratory parameters can effectively predict colorectal cancer patient outcomes.

The prognostic value of ANPG in CRC is rooted in the pathophysiological interplay between nutritional status and systemic inflammation in cancer progression. As a negative acute-phase reactant, albumin serves as a dual biomarker reflecting both nutritional reserve and systemic inflammatory response. Hypoalbuminemia is associated with impaired immune function, increased risk of infection, and reduced tolerance to therapy (20, 25). Concurrently, neutrophilia reflects an exaggerated systemic inflammatory response, which promotes tumor progression through multiple mechanisms, including angiogenesis induction, extracellular matrix remodeling, and suppression of antitumor immunity (22, 23). By integrating these two clinically relevant parameters, the ANPG system provides a comprehensive assessment of the host inflammatory-nutritional axis, demonstrating greater prognostic accuracy than individual markers or other composite scores.

The current study builds on these observations by confirming that ANPG serves as an independent predictor of clinical outcomes in a large CRC cohort, outperforming multiple established inflammatory-nutritional indices. Specifically, comparative analysis revealed that ANPG exhibited significantly superior discriminative capability (AUC = 0.637) relative to the neutrophil-to-lymphocyte ratio (NLR, 0.574), platelet-to-lymphocyte ratio (PLR, 0.519), systemic immune-inflammation index (SII, 0.537), and fibrinogen-NLR (F-NLR, 0.591) (all P<0.05 by DeLong’s test). Notably, ANPG incorporates only two routinely measured preoperative parameters—serum albumin and neutrophil counts—offering distinct clinical utility without necessitating additional diagnostic expenditures. Emerging evidence from randomized controlled trials suggests that perioperative nutritional intervention may mitigate postoperative complications in patients with elevated inflammatory markers (26). Thus, ANPG represents a practical clinical tool for identifying high-risk patients who may benefit from intensive preoperative nutritional optimization. Specifically, patients classified as ANPG grade 2 (exhibiting concurrent hypoalbuminemia and neutrophilia) might experience reduced complication rates following targeted nutritional support, although this hypothesis requires prospective validation.

In contrast to composite scoring systems that rely on multiple biomarkers, ANPG offers enhanced operational simplicity and greater feasibility in resource-constrained settings. Its prognostic superiority likely stems from the simultaneous evaluation of nutritional status and systemic inflammation, thereby providing a more comprehensive assessment of host-tumor dynamics. This streamlined methodology renders ANPG particularly suitable for implementation in primary care facilities or institutions with limited diagnostic infrastructure, facilitating broader clinical applicability.

Notably, patients with elevated ANPG scores were significantly older (P = 0.001). This association may be explained by age-related declines in nutritional status and increased systemic inflammation—both recognized contributors to tumor progression (27, 28). Aging is linked to reduced albumin synthesis, dysregulated immune function, and chronic low-grade inflammation (termed inflammaging), which may collectively lead to higher ANPG scores and poorer clinical outcomes in older individuals. Furthermore, This study’s univariate Cox regression analysis revealed that patients receiving postoperative adjuvant chemotherapy had a significantly higher risk of mortality compared to those who did not undergo chemotherapy. However, this association disappeared after multivariate adjustment. Although both analyses showed consistent directionality of association (HR>1.0), the effect size was significantly reduced and lost statistical significance after adjustment, indicating that the initial association was primarily confounded by prognostic factors such as tumor stage rather than reflecting a true detrimental effect of chemotherapy. This phenomenon—where chemotherapy appears harmful in univariate analysis but becomes non-significant after adjustment for stage—represents a classic example of confounding by indication. Specifically, patients with advanced-stage disease are more likely to receive chemotherapy, yet they inherently had poorer prognoses. In this study, the chemotherapy recipients had a significantly higher proportion of stage III cases compared to the non-chemotherapy group (48.6% vs. 20.2%), suggesting greater tumor burden and more advanced disease progression, which explains the primary reason for the observed association between chemotherapy and worse prognosis in univariate analysis.

These findings are consistent with previous prognostic studies in colorectal cancer. Zhang et al. (29) demonstrated in their study on an inflammatory-nutritional prognostic model for colorectal cancer that the 5-year overall survival rate was significantly lower in patients receiving adjuvant therapy (75.3%) compared to those not receiving adjuvant therapy (88.7%, P = 0.010) in univariate analysis, but adjuvant therapy lost independent prognostic significance after multivariate adjustment. Similarly, Sun et al.’s study on non-small cell lung cancer that postoperative radiochemotherapy was a prognostic factor for overall survival (OS) in univariate analysis (Yes vs No:HR=1.535, 95%CI:1.271–1.854, p<0.001), yet it failed to serve as an independent prognostic factor for OS in multivariate analysis when it was analyzed with neutrophil or ANPG (24). It is important to emphasize that these results do not negate the clinical value of chemotherapy in cancer treatment, but rather highlight the need to consider other factors when evaluating chemotherapy’s impact on clinical outcomes.

As a well-established tumor marker in CRC, carcinoembryonic antigen (CEA) is closely related to tumor burden and recurrence risk, and its role in prognostic models deserves attention (30). Our comparative analysis demonstrated that incorporation of CEA improved the nomogram’s discriminative accuracy, increasing the concordance index (C-index) from 0.800 to 0.806, thus confirming its incremental prognostic value. This finding aligns with existing evidence indicating that conventional tumor markers can complement inflammatory and nutritional biomarkers to enhance predictive model performance (31, 32). Although CEA did not reach statistical significance in multivariate analysis (P = 0.075), its inclusion in the model is justified by the observed improvement in predictive capability and its established clinical relevance. This approach underscores the principle that prognostic models should balance statistical rigor with clinical meaningfulness (33).

Based on the above clinical correlation analysis, we further verified the independent prognostic value of ANPG using multivariate analysis. The results showed that although the fibrinogen-to-albumin ratio (FAR) was significantly associated with prognosis in univariate analysis, its prognostic significance was lost after adjustment for ANPG and other covariates, suggesting that ANPG captures more comprehensive prognostic information than FAR. This finding contrasts with some prior reports (34, 35), but aligns with a recent meta-analysis indicating inconsistent prognostic value of FAR in colorectal cancer (16). More importantly, ANPG remained an independent prognostic factor beyond traditional clinical variables including age, CA19-9, histological subtype, CEA, and TNM stage, highlighting its important incremental value in risk stratification for CRC patients.

The developed nomogram serves as a clinically applicable decision-support tool, demonstrating strong predictive performance with a concordance index (C-index) of 0.806. It exhibits superior calibration compared to existing prognostic nomograms for colorectal cancer that integrate TNM staging and inflammatory biomarkers (36), offering more accurate individualized survival predictions than conventional staging systems alone. Time-dependent receiver operating characteristic (ROC) analysis confirmed enhanced discrimination, with consistently higher area under the curve (AUC) values at 1-, 3-, and 5-year follow-up timepoints. Decision curve analysis further validated its clinical utility, revealing greater net benefit across relevant threshold probabilities, supporting its potential to guide treatment decisions and optimize resource utilization. These results have significant clinical implications, as the nomogram may facilitate more precise identification of high-risk patients who could benefit from intensified surveillance, adjuvant therapy, or nutritional interventions.

This study has several key strengths. First, the relatively large sample size (n = 660) and extended follow-up duration (median 2442 days) provide robust statistical power and reliable survival estimates. Second, strict inclusion criteria—limited to R0-resected cases and exclusion of patients with confounding conditions—help minimize bias. Third, systematic comparison with multiple established inflammatory and nutritional markers highlights the superior prognostic performance of the ANPG score. Fourth, comprehensive statistical validation methods, including bootstrap resampling and time-dependent ROC analysis, strengthen the reliability and generalizability of the findings.

Several limitations of this study must be acknowledged. First, as a single-center retrospective investigation, the potential for selection bias and limited external validity necessitate validation through multicenter studies. Second, the cutoff values for albumin and neutrophil counts were derived from this specific cohort, warranting further evaluation of their applicability in broader populations. Third, the absence of comprehensive molecular profiling—including assessment of microsatellite instability (MSI) status—precluded definitive subgroup analyses based on molecular phenotype, a critical consideration given the well-documented molecular heterogeneity of colorectal cancer (37–39). Emerging evidence suggests that patients with MSI-high (MSI-H) colorectal cancer often exhibit heightened systemic inflammation and impaired nutritional status compared to those with microsatellite stable (MSS) tumors (38). Given that the ANPG score reflects inflammatory-nutritional status, its prognostic performance may vary according to molecular subtypes, potentially demonstrating subtype-specific differences. Future prospective studies incorporating comprehensive molecular profiling, including MSI status assessment, are required to determine whether the predictive utility of the ANPG score remains consistent across colorectal cancer subtypes. Such studies will clarify whether ANPG serves as a universal prognostic indicator or whether its application should be refined within the framework of precision oncology, potentially requiring subtype-specific cutoffs or interpretations.

## 5 Conclusion

The ANPG score, derived from routinely available laboratory parameters, provides a practical and accessible tool for prognostic stratification in colorectal cancer patients, particularly in settings where C-reactive protein (CRP) measurement is not routinely performed. Its comparable performance to established inflammatory-nutritional scores underscores the potential of simplified biomarker models in clinical oncology while the developed nomogram provides a clinically valuable tool for individualized survival prediction and risk stratification.

## Data Availability

The original contributions presented in the study are included in the article/supplementary material. Further inquiries can be directed to the corresponding author/s.
